# Dr. Archibald McNiel Bevier

**Published:** 1913-04

**Authors:** 


Dr. Archibald McNiel Bevier, Buffalo 1866, for 45 years a practitioner of Owasco, N. Y., died at his home Feb. 19 of arterio-sclerosis, aged 71.

